# Cognitive Trajectories over 13 Years in Schizophrenia Spectrum Disorders: Addressing Selective Dropout Bias

**DOI:** 10.1093/schbul/sbag162

**Published:** 2026-10-30

**Authors:** Maria Lee, Granville J Matheson, Ellen E Lee, Sara Holmqvist, Håkan Nyman, Ingrid Agartz, Alison R Hwong, Erik G Jönsson

**Affiliations:** Centre for Psychiatry Research, Department of Clinical Neuroscience, Karolinska Institutet & Stockholm Health Care Services, 171 77, Stockholm, Sweden; Department of Medical Epidemiology and Biostatistics, Karolinska Institutet, 171 77, Stockholm, Sweden; Department of Psychiatry and Behavioral Sciences, Stanford School of Medicine, Stanford, CA 94305-5717, United States; Centre for Psychiatry Research, Department of Clinical Neuroscience, Karolinska Institutet & Stockholm Health Care Services, 171 77, Stockholm, Sweden; Department of Psychiatry, University of California San Diego, La Jolla, CA 92093, United States; Centre for Psychiatry Research, Department of Clinical Neuroscience, Karolinska Institutet & Stockholm Health Care Services, 171 77, Stockholm, Sweden; Department of Clinical Neuroscience, Karolinska Institutet, 171 77, Stockholm, Sweden; Centre for Psychiatry Research, Department of Clinical Neuroscience, Karolinska Institutet & Stockholm Health Care Services, 171 77, Stockholm, Sweden; Department of Psychiatric Research, Diakonhjemmet Hospital, 0319 Oslo, Norway; Centre of Precision Medicine, Institute of Clinical Medicine, University of Oslo, 0318 Oslo, Norway; Department of Psychiatry and Behavioral Sciences, Stanford School of Medicine, Stanford, CA 94305-5717, United States; VA Palo Alto Health Care System, Palo Alto, CA 94304-1207, United States; Centre for Psychiatry Research, Department of Clinical Neuroscience, Karolinska Institutet & Stockholm Health Care Services, 171 77, Stockholm, Sweden; Institute of Clinical Medicine, Division of Mental Health and Addiction, University of Oslo, 0424 Oslo, Norway

**Keywords:** psychosis, longitudinal, cognition, attrition, cognitive trajectories over 13 years in schizophrenia: addressing selective dropout bias

## Abstract

**Background and Hypothesis:**

The longitudinal course of cognitive functioning in schizophrenia is debated. Previous longitudinal studies have reported selective attrition. We aimed to analyze cognitive trajectories over 13 years among patients with schizophrenia spectrum disorders (SSDs) in comparison with controls, while accounting for dropout bias, and explored baseline predictors of cognitive outcome.

**Study Design:**

Cognition was assessed at study start, 5-year, and 13-year follow-up in 108 participants with schizophrenia spectrum disorder and 85 controls of similar age. Linear Mixed Effects models were used to model trajectories over time, with inverse probability weights to account for non-random dropout in patients. Predictors of cognitive function at 13 years were explored using linear regression.

**Study Results:**

Retention was 51% for patients and 65% for controls. At study start, patients who dropped out performed worse on most cognitive tasks and were significantly older (4.5 years) than completers. In primary inverse probability of attrition weight–weighted analyses, patients scored lower than controls across nearly all cognitive domains (cognitive composite: β = −0.91 SD [95% CI −1.42, −0.39]) but their trajectories did not significantly differ from those of controls. Higher age predicted worse cognitive function. Illness duration was an independent negative predictor in patients. Study start cognition predicted 13-year follow-up cognition in patients.

**Conclusions:**

Although cognitive performance of patients was lower than that of controls at study start, their change in performance over 13 years did not differ from the controls. Patients who dropped out were more cognitively impaired, highlighting risk of attrition bias. The robustness of findings across weighted and unweighted analyses strengthens confidence in the observed trajectories.

## Introduction

Cognitive dysfunction in schizophrenia is common and widespread. There is often significant cognitive impairment already prior to the onset of psychosis.^1^ Studies in the longitudinal course of cognitive function have produced mixed findings. Some literature find that cognition stays stable a decade or more after illness onset,^2–6^ although there are also reports of further deterioration^7^^,^^8^ or even improvement^9^ in the long-term. A recent meta-analysis^10^ points to stability over time, but the majority of included studies followed first episode psychosis patients, where longstanding care needs may vary. What trajectories look like in the long-term for individuals who do need continued treatment is a clinically relevant question. The only two studies on individuals in long-term treatment with follow-up durations over ten years in the meta-analysis point primarily to stable cognition.^2^^,^^11^ Mohn et al.,^12^ has since published data on 20-year follow-up in those with schizophrenia in long-term treatment, also indicating stability except for verbal ability. As for domain-specific trajectories, verbal learning and memory appear particularly vulnerable in some studies,^7^^,^^12^^,^^13^ with decline or reduced practice effects observed in this domain, though this has not been consistently replicated.^3^^,^^9^^,^^14^

Specifically in older ages, when individuals with schizophrenia enter mid-to-late life, there are reports indicating continued decline. Friedman et al.^15^ demonstrated accelerated cognitive decline after age 65 in a sample of institutionalized patients with schizophrenia, and Jonas et al.^8^ found evidence of further cognitive deterioration after age 50 years in their very large (*N* = 428) longitudinal sample. These findings have been interpreted as supporting the accelerated aging hypothesis,^11^^,^^12^ where schizophrenia can partly be seen as a disorder of accelerated aging, ^16^^,^^17^ resulting in premature cognitive decline and development of long-term medical conditions typically associated with older age, such as cardiovascular disease and type 2 diabetes.^18^^,^^19^ However, the literature is mixed, with other longitudinal studies finding similar rates of decline in patients and controls,^20^ suggesting parallel aging processes. If schizophrenia does involve accelerated aging, one would expect steeper cognitive decline in patients relative to controls, particularly at older ages.

Scarcity of longitudinal data is partly due to inherent challenges in following this group over time,^21^ where low level of functioning and amotivation may play a role.^22^ Given these challenges, the attrition rate in longitudinal studies is typically high (40%-50%^2^^,^^3^^,^^7^^,^^13^), and concerns have been raised about how this attrition-related selection bias affects the results and conclusions drawn.^2^ It is possible that survivor bias plays a major role in skewing findings, with the healthiest and most engaged patients continuing to be followed in long-term studies. Furthermore, studies without a control group may not clearly differentiate between normal aging and psychosis-related decline.

Cognitive trajectories in schizophrenia are also known to be heterogeneous,^23^ and variability between individuals has been reported to increase over time.^6^ Factors associated with long-term (10 years or longer) cognitive outcome or cognitive change are not clear and have not been consistently replicated (for a review, see Starzer et al.^2^). Individual studies have identified age of onset,^24^^,^^25^ sex,^24^ and premorbid intelligence quotient (IQ)^4^ as predictors. Baseline cognitive function^25^^,^^26^ and avoiding relapses^5^ have also been associated with better long-term cognitive outcomes, while more severe psychotic symptoms at baseline have been linked to greater decline in memory domains.^7^

In this study, we followed a cohort of patients with clinically diagnosed schizophrenia spectrum disorders (SSDs) and controls over a 13-year period. Our aim was to analyze the general trajectories of cognition, whether these differed between the two groups and which specific baseline factors were associated with worse decline. In addition, given the history of attrition in these long-term studies of people with schizophrenia, we aimed to control for potential dropout bias in our analyses.

## Methods

### Participants

Patients with SSD and controls were recruited as part of the HUman Brain INformatics (HUBIN) study, at the Karolinska Institutet in Sweden, which has been described previously in detail.^27^^,^^28^ HUBIN is a multi-modal longitudinal study of individuals with SSD, including repeated magnetic resonance imaging (MRI), clinical interviews, and cognitive function measured at study start, at 5 years, and at 13 years. Brain imaging results from this cohort at 13-year follow-up have been reported separately.^29^^,^^30^ Cognitive results from the 5-year follow-up were reported in Ekerholm et al.^31^ The study was approved by the Stockholm regional Ethical Review Authority (ref. no 2009/1465-31/3). All participants provided written informed consent. HUBIN included both patients with SSD, their relatives, and unrelated controls. In this analysis, only individuals with SSD and controls were analyzed, and only those with cognitive test results from at least the first assessment (at study start) were retained.

Patients with SSD were recruited from outpatient psychiatric clinics in northern Stockholm County, Sweden. Individuals with a diagnosis of schizophrenia, or schizophreniform psychosis, were asked to participate. Exclusion criteria were a history of head trauma with loss of consciousness for more than 5 min and somatic disorders affecting brain function. Controls were either drawn from a national population register or recruited among students or hospital staff members who had previously participated in clinical studies at the Karolinska Institutet. In addition to the exclusion criteria for patients, controls were excluded if they had a psychotic disorder diagnosis. Participants were included between 1999 and 2003, and the initial assessments took place in the same time period. Diagnostic interviews and symptom assessments were conducted by the senior author, E.G.J., an experienced psychiatrist, both at study start and at subsequent follow-ups.

In sum, the sample at study start consisted of 108 individuals with SSD and 85 controls.

### Cognitive Test Battery

The battery of tests used was the Cognitive Performance Indicator (CPI), a semi-computerized battery meant to provide an overview of cognitive function in schizophrenia.^32^ Tests included in the battery are: Rey Auditory Verbal Learning Test (RAVLT, measuring verbal learning and memory), Letter Number Sequencing (LNS, a test of working memory), Continuous Performance Test—Identical Pairs (CPT-IP, measuring attention and vigilance), Trail Making Test A and B (TMT-A/TMT-B, measuring processing speed, and cognitive flexibility, respectively), Wechsler Adult Intelligence Scale-III Vocabulary (measuring verbal ability and used as a proxy of pre-morbid intelligence) and the 64 card version of the Wisconsin Card Sorting Test (WCST, measuring set-shifting failure).

The outcome measures selected for analysis were: RAVLT total recall (total number of words recalled over the trial, measuring verbal learning), RAVLT retention (number of words recalled after 20 min), LNS (number of correctly reproduced sequences), CPT-IP *d*′ (an index of signal-to-noise discrimination), TMT-A (seconds to complete), TMT-B (seconds to complete), WCST Perseverative Errors (number of errors).

Upon inspection, 8 of the CPT-IP values were outside the plausible range (eg., 8000 instead of plausible range −4.24 to +4.24^33^) and these values were therefore removed. For TMT, some test performances (*N* = 1 for TMT-A, *N* = 7 for TMT-B) were slower than the recommended allotted time (more than 180 and 300 s respectively).^34^ These values were retained, as this likely reflect very poor functioning rather than malingering or inability to comprehend instructions. TMT scores were logarithmically transformed for the analyses, which reduces the influence of extreme values on model estimates.

From the cognitive test battery, a cognitive composite score was calculated, using control mean and standard deviations at baseline to calculate *z*-scores. The distributions of test scores were visually inspected and three tests (TMT-A, TMT-B, WCST Perseverative Errors) were positively skewed rather than normally distributed. These scores were logarithmically transformed and reversed (as smaller values indicate better performance in these tests). *Z*-scores were then averaged, to construct a composite score. In addition to this, more clinically interpretable cognitive composite, a principal component analysis (PCA) was also performed in order to get a single value for cognition to use in inverse probability of attrition weights (IPW)-weighting.

### Statistical Analysis

For the dropout analysis, we considered patients and controls who returned for any follow-up as completers, and those who only attended the baseline testing as dropouts. This definition was motivated by the fact that follow-up recruitment was more successful at the 13-year assessment than at the 5-year assessment, due to greater scheduling flexibility at the later timepoint. Attendance at any follow-up was therefore considered to more meaningfully capture ability and willingness to participate, than attendance at 13-year follow-up specifically. Dropouts and completers were compared on baseline demographics and cognition using *t-*test or chi-square tests.

In order to model cognitive change over time, we used Linear Mixed Effects (LME) models. Models were built in consecutive steps, starting with random intercept for all participants, then adding fixed effects, in order: (1) group (patient or control), (2) timepoint, (3) timepoint * group interaction, (4) illness duration at start of study, (5) age (centered), and (6) sex, as predictor variables. Akaike information criterion (AIC) and Bayesian information criterion (BIC) values were used to compare model fit at each step as model complexity increased. See Supplementary methods for details on full LME model specification and quality check.

We modeled change using timepoint (study start, 5-year, 13-year) as a categorical predictor, with age (centered, time-varying) included as a continuous covariate. This approach allows us to examine average change across measurement occasions while controlling for age-related differences in performance. Timepoint captures effects associated with repeated assessment (eg, practice effects, measurement occasion-specific factors), while age captures the relationship between current age and cognitive performance. Additionally, timepoint serves as an anchor for the dropout analysis. Illness duration at study start (non-time-varying) captures how long patients had been living with SSD at study entry—whether patients with longer illness histories show poorer cognitive function throughout the study period. Although age and illness duration share a mathematical relationship (illness duration = age − age of onset), they represent two different concepts: age captures the effect of getting older on cognitive performance, whereas illness duration at baseline captures effects of living with schizophrenia (such as medication exposure, lifestyle changes due to illness) above and beyond current age. Age was centered to reduce multicollinearity with illness duration, and Variance Inflation Factor (VIF) values for all main predictors in the final model were below 5 (see Supplementary Methods for details). Antipsychotic medication status was not included as a covariate, as the majority of patients were using antipsychotic medication at study start (89%), providing insufficient variation for inclusion. Data on cumulative medication exposure over the whole study period was not available.

As the number of observations per participant were limited (median = 2), we were unable to estimate individual age-related trajectories using random slopes. Exploratory analyses with three-way interaction (age × group × timepoint) were also run, in order to examine whether age moderated group trajectory differences. Age distribution across timepoints for the two groups can be seen in Figure S1.

### Inverse Probability Weighting

In order to account for the proposed effects of attrition, we used IPW in our models. This method adjusts the weight of participants based on the likelihood of remaining in the study, where those more similar to participants who are lost to follow-up are assigned greater weight.^35^ Weighting was informed by the dropout analysis, and only assigned to patients as there were no statistically significant dropout differences of interest in the control group (global assessment of function [GAF] differences were not considered clinically meaningful, as both control groups had high scores indicating good functioning in all areas, see Results below). Controls were all assigned a weight of 1. Models using IPW are presented as the main analysis but sensitivity analyses were conducted without these weights (see Tables S5–S12).

Exploratory analyses investigating change in cognition and specific predictors of worse function were conducted in patients only, and in those who provided data both at study start and 13-year follow-up (*N* = 45 controls and 45 patients). First, outcome at 13-year follow-up was used as the dependent variable in a linear regression with cognition at study start, age of onset, premorbid IQ, and gender as predictors. Second, change scores were derived by subtracting the cognitive composite score at 13 years from the cognitive composite score at study start. A categorical definition of poor outcome was used, assigning those with a change score below the 25% percentile for patients (−1.05 SD) as poor performers. This value corresponded to a more severe decline than for the bottom 10th percentile in controls (−0.95 SD). The poor performing patients hence fall well below what would be considered typical age-related decline over 13 years.

All statistical analyses were conducted in R^36^ version 4.4.2 (2024-10-31). LME models were calculated using the lme4^37^ and lmerTest^38^ packages. Given the exploratory nature of the study, no corrections for multiple comparisons were applied.

## Results

Table 1 presents descriptive statistics of the participants at study start. Controls had significantly higher level of function, were more likely to be working, and had more years of education compared to patients. Sex composition at study start was also significantly different between groups, where controls were more likely to be female. There was a wide range in duration of illness and age of onset for participants with SSD, and the vast majority were on antipsychotic medication at the start of the study.

**Table 1:** Demographic Information on Full Sample at Baseline.

	Patients (*N* = 108)	Controls (*N* = 85)	*t*/*χ* ^2^	Df	*P*
Age, mean (SD), years	43.4 (9.0)	45.1 (7.4)	1.44	190.4	.151
Age, range, year	25-–70	22-57			
Sex, F/M, *N* (%)	30 (28)/78 (72)	38 (45)/47 (55)			.016
Working status, yes/no	26 / 75 *N* = 101	83 / 2			<.001
Education, mean (SD), year	12.1 (2.8) *N* = 98	14.1 (2.3) *N* = 83	4.55	168.7	<.001
GAF, mean (SD), score	48.2 (9.3) *N* = 90	85.7 (7.3) *N* = 81	29.48	165.9	<.001
Diagnosis, *N* (%)					
Schizophrenia	79 (73)				
Schizoaffective disorder	13 (12)				
Psychotic disorder NOS	12 (11)				
Substance-induced	1 (1)				
No information	3 (3)				
Age of onset, mean (SD), year	23.9 (6.2) *N* = 91				
Age of onset, range, year	16-44				
Duration of illness, mean (SD), year	18.6 (9.1) *N* = 105				
Duration of illness, range, year	0-44				
Medication status *N* (%)					
Antipsychotics use	96 (89)				
No antipsychotics	8 (7)				
No information	4 (4)				

Working status: working, working/normal retirement, not working, unemployed/sickness absence/disability pension (for more detailed information, see Table S1); GAF, global assessment of function.

Of the 108 patients who underwent cognitive testing, 36 came back for testing at the 5-year follow-up and 45 for the 13-year follow-up. For the 85 controls at study start, 34 returned for the 5-year follow-up and 45 for the 13-year follow-up. Follow-up recruitment was more successful at the 13-year time-point compared to the 5-year follow-up timepoint, due to logistics and scheduling issues. Although only 26 patients and 24 controls had complete data from all three timepoints, the percentage retained from study start to 13-year follow-up (ie, participants with at least baseline and 13 year assessment) was 41.6% for patients and 53.9% for controls.

### Dropout Analysis

The dropout analysis focused on whether participants returned to any follow-up assessment (5 or 13-year). Out of 108 participants with SSD who completed cognitive testing at study start, 55 returned for any follow-up (51% retention). Out of the 85 healthy participants who were tested at study start, 55 returned for any follow-up (65% retention). Reasons for non-participation at 13-year-follow up were available and are detailed in Table S2. The most common reasons for patients were administrative (not contacted due to scheduling or MRI participation; *N* = 22), not reachable (*N* = 12), and death (*N* = 12) (see Table S3 for descriptive comparison of baseline data on patients who had died at 13 year follow-up, vs. those still alive). No controls had died by follow-up. Among controls, the most commons reasons were not reachable (*N* = 12) and logistical factors such as lack of time or relocation (*N* = 10).

Among patients, those who dropped out were significantly older (mean 4.5 years) and less likely to be working at study start. There were no statistically significant differences in symptom burden (Scale for the Assessment of Positive Symptoms (SAPS), *P* = .263; Scale for the Assessment of Negative Symptoms (SANS), *P* = .456), age of onset (*P* = .367), or years of education (*P* = .133). Cognitive scores were significantly lower for nearly all tasks in the dropout group, apart from the test of attention and vigilance (CPT-IP) and set-shifting (WCST-PE). In the control group the only difference between groups was that those who dropped out had significantly lower ratings of GAF. There were no significant differences in cognitive function at study start for controls. For details, see Table 2.

**Table 2:** Demographic Information and Cognitive Function at Study Start in Those Who Completed Any Follow-Up (5-Year, 13-Year, or Both) vs. Those Who Dropped Out.

	Patients follow-up mean (SD)	*N*	Patients drop out mean (SD)	*N*	*P*		Controls follow-up mean (SD)	*N*	Controls drop out mean (SD)	*N*	*P*	
Age, years	41.2 (7.9)	55	45.7 (9.4)	53	**0.009**		44.4 (7.8)	55	46.5 (6.7)	30	0.195	
Sex, female/male, *N*	11 / 44	55	19 / 34	53	0.086		23 (F) / 32 (M)	55	15 (F) / 15 (M)	30	0.501	
Working status, yes/no, *N*	20 / 33	53	6 /42	48	**0.006**		53/2	55	30/0	30	0.538	
GAF, score	49.8 (10.0)	44	46.7 (8.5)	46	0.125		87.2 (5.5)	52	83.1 (9.3)	29	0.035	
Premorbid IQ	91.8 (19.2)	55	86.2 (20.7)	53	0.150		102.9 (15.2)	55	100.7 (16.7)	30	0.554	
Education, years	12.5 (2.6)	52	11.7 (2.9)	46	0.133		14.1 (2.8)	54	14.0 (3.3)	29	0.896	
Age of onset, years	24.1 (4.8)	55	25.4 (9.0)	50	0.367		NA		NA			
Illness duration, years	17.1 (7.7)	55	20.3 (10.3)	50	0.080		NA		NA			
Positive symptoms (SAPS), score	10.7 (8.2)	31	13.9 (11.1)	27	0.236		NA		NA			
Negative symptoms (SANS), score	32.6 (23.5)	31	28.4 (19.0)	28	0.456		NA		NA			
**Baseline cognition**					***P***	**Cohen’s *d***					***P***	**Cohen’s *d***
RAVLT total	45.5 (11.1)	55	37.9 (12.3)	53	**.001**	**(−0.65)**	55.5 (6.1)	55	53.7 (6.5)	30	.213	(−0.29)
RAVLT retention	9.3 (3.7)	55	7.7 (3.7)	53	**.029**	**(−0.42)**	12.5 (2.1)	54	12.0 (2.3)	30	.291	(−0.25)
CPT-IP	0.6496 (0.715)	53	0.659 (0.658)	53	.939	(0.01)	1.176 (0.650)	55	1.129 (0.628)	30	.747	(−0.07)
TMT-A	32.3 (11.8)	55	47.6 (36.1)	53	**.005**	**(0.58)**	24.3 (7.9)	55	21.8 (6.0)	30	.105	(−0.34)
TMT-B	91.3 (48.8)	55	136.6 (103.1)	53	**.005**	**(0.57)**	56.4 (18.3)	55	67.6 (39.1)	30	.146	(0.41)
LNS	9.3 (2.6)	55	7.7 (3.4)	53	**.006**	**(−0.54)**	11.1 (2.4)	55	10.8 (1.8)	30	.503	(−0.14)
WCST-PE	11.0 (7.6)	55	13.9 (8.9)	53	.082	(0.34)	7.1 (4.1)	55	8.1 (5.1)	30	.331	(0.24)

Bold *P* values indicate statistical significance (α = 0.05). Abbreviations: GAF, global assessment of function; NA, not applicable; SAPS, Scale for the Assessment of Positive Symptoms; SANS, Scale for the Assessment of Negative Symptoms; RAVLT, Rey Auditory Verbal Learning Test; CPT-IP, Continuous Performance Test—Identical Pairs Version; TMT, Trail Making Test; LNS, Letter Number Sequencing; WCST-PE, Wisconsin Card Sorting Test—Perseverative Errors.

### Analysis of Change over Time

As mentioned, given the selective dropout in the patient group specifically, we decided to account for this in subsequent analytic models using IPW.

#### IPW Model

A PCA was applied to the cognitive data for use in the adjusted weights. The first component explained approximately 60.9% of the total variance and loaded on all cognitive tests (see Table S4).

To calculate weights, we first estimated the probability of retention for each participant in the SSD group using a logistic regression model (propensity score model) with follow-up status as the outcome; participants attending baseline only were assigned a weight of 1, while those with at least one follow-up assessment were weighted by the inverse of their predicted probability of retention (1/*p*). Variables were selected based on their association to dropout in preliminary analyses (see Table 2) and theoretical relevance to cognitive outcomes.^39^ Age, gender, and cognition (using the first principal component of the PCA) at study start were included; employment status was excluded to avoid positivity violations (given the modest sample size). No trimming was applied as the distribution of weights was narrow (range: 1.18 to 3.51, mean 1.86), indicating no extreme weights that would risk inflating variance of effect estimates.^40^^,^^41^

#### LME Models

The final model for the neurocognitive composite included random intercept, fixed effect of group, timepoint, age (centered), baseline illness duration, sex, and a group × timepoint interaction effect. The last step, adding age (centered) to the model, significantly improved model fit (χ^2^(1) = 12.89, *P* < .001, AIC/BIC with age: 885/927, vs AIC/BIC without: 896/934).

For the neurocognitive composite, individuals with SSD started out lower—scoring 0.91 standard deviations below controls at study start (β = -0.91, *P* < .001), when controlling for age, sex, and illness duration. Being older was associated with worse cognitive function across all assessments, with scores 0.03 standard deviations lower per year of age (*P* < .001). For patients, longer illness duration at study start was independently associated with lower scores (β = −0.03 per year, *P* = .016). Sex did not have a significant impact on estimates of cognition (*P* = .45). The interaction effect between timepoint and group was not significant (both *P* > .76), indicating that cognitive trajectories in the two groups were similar. Main effect of timepoint was also non-significant (both *P* > .28), implying no additional change in performance across timepoints after accounting for age.

The random intercepts for participants (SD = 0.70) indicated substantial between-subject variability, while the residual (SD = 0.67) reflected within-person fluctuations over time and measurement noise.

A summary of the final model can be found in Table 3 and is visualized along with raw scores in the raincloud plot in Figure 1H.

**Table 3:** Final Linear Mixed Effect Model Summary for Cognitive Composite Score

Fixed effects	Estimate	SE	*P* value
Intercept	−0.020	0.130	.878
Group (Control as reference)	−0.906	0.261	<.001^***^
Timepoint 5 years	0.162	0.151	.285
Timepoint 13 years	0.189	0.172	.276
Illness duration at study start	–0.028	0.011	.016^*^
Age (centered)	−0.033	0.009	<.001^***^
Sex: Male	−0.102	0.134	.446
Group by Time, Timepoint 5 years: SSD	0.045	0.184	.808
Group by Time, Timepoint 13 years: SSD	−0.051	0.166	.760
**Random effects**	**SD estimate**		
Intercept	0.699	–	–
Residual variance	0.671	–	–
Model fit: AIC = 885.4, BIC = 927.9	–	–	–

**Figure 1:**
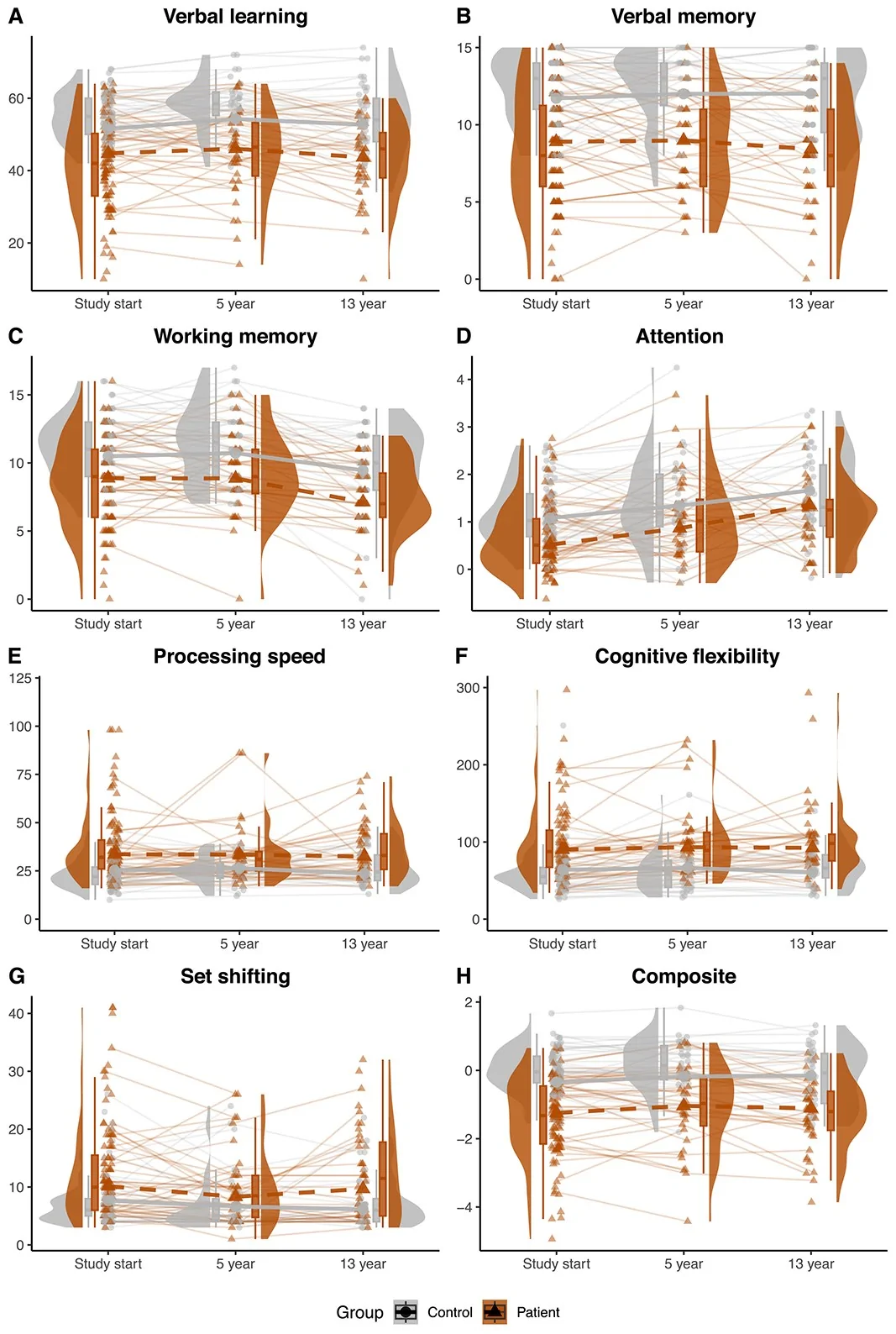
Raincloud Plots of Cognitive Trajectories over 13 Years. Patients in Orange (Triangles and dashed lines) and Controls in Gray (Circles and solid lines). Raw Data Shown as: Individual Points, Density Distributions, and Boxplots. Superimposed Lines and Points Show Estimated Marginal Means from Inverse Probability Weighted Linear Mixed Effects models. Verbal Learning, RAVLT Total; Verbal Memory, RAVLT Retention; Working Memory, LNS; Attention, CPT-IP; Processing Speed, TMT-A; Cognitive Flexibility, TMT-B; Set-Shifting, WCST-Perseverative Errors; Composite, Cognitive Composite. Panels (A–D) Higher Scores Indicate Better Performance. Panels (E–G) Lower Scores Indicate Better Performance. Panel (H) Values in *z*-Scores, Higher Value Indicate Better Performance.

In the domain specific models (Figure 1A–G) there was a significant group effect in all domains (except for set-shifting, *P* = .077) where controls consistently outperformed patients at study start (Tables S6–S12). Older age at assessment was a negative predictor in all domains except for working memory. Longer illness duration at study start had an additional, negative effect, beyond age and group on performance in verbal learning and processing speed. Male sex was significantly associated with lower scores in verbal learning and memory.

There was a significant effect of timepoint at year 13 in the domains of working memory and attention, with estimates indicating a decline across both groups, beyond the effects of age, in working memory (β -1.03, *P* = .046) at the 13-year follow-up, and an improvement across groups in attention (β = 0.58, *P* ≤ .001) at the same timepoint. No timepoint × group interaction was statistically significant, indicating that the two groups followed similar trajectories. For verbal learning (RAVLT total), there was a non-significant trend toward diverging trajectories (Patient × 5 years: β = −1.53, *P* = .40; Patient × 13 years: β = −2.29, *P* = .17).

### Predictors Associated with Worse Trajectories

Higher cognitive function at study start (measured by the cognitive composite, β = 0.454, p = 0.029) predicted better outcomes at 13-year follow-up (see Table 4). The model fit (*R*^2^ = 0.27, Adjusted *R*^2^ = 0.20) indicates that the model explains 27% of the variance in cognitive composite at 13 years.

**Table 4:** Summary of Linear Regression Model Predicting Cognitive Composite at 13 Year.

Predictor	Estimate (*β*)	Std. error	*t* value	*P* value	95% CI
(Intercept)	−3.475	1.11	−3.12	.003^**^	−5.73 to −1.23
Age of onset	0.033	0.03	1.16	.252	−0.02 to 0.09
Sex (male)	0.206	0.34	0.60	.550	−0.48 to 0.90
Premorbid IQ	0.017	0.01	1.81	.077	−0.00 to 0.04
Baseline cognition	0.454	0.20	2.26	.029^*^	0.05 to 0.86

The categorical model, looking at the bottom quartile of the patient population (corresponding to worse cognitive decline than 90% of controls), was unable to identify any statistically significant predictors (all *P* > .113) (see Table S13). Raw cognitive scores for those who completed testing at study start and 13-year follow-up are in Table S14, and demographics and cognitive raw scores for complete cases are in Tables S15 and S16.

#### Sensitivity Analysis of LME Models Using IPW versus Unweighted LME Models

Sensitivity analyses comparing IPW-weighted to unweighted models showed substantively similar results across domains. Full comparisons can be found in Tables S5–S12.

#### Exploratory Analysis of Age Effects on Group Trajectory

No consistent pattern emerged across cognitive domains when three-way interaction of age × time × group was added to models. The cognitive composite showed a nominally significant three-way interaction for patients at the 13 year follow-up (β +0.048 [95% CI +0.01 to +0.09], *P* = .022), but this was not replicated in individual domains. The cognitive composite finding did not survive False Discovery Rate (FDR) correction across cognitive domains, and the three-way models did not improve statistical fit (see Table S17).

## Discussion

In this longitudinal study of participants with SSD and controls, we found that dropout was not random for patients with SSD—it was predicted by age and cognitive function at study start. In order to account for this, we analyzed cognitive trajectories using inverse probability weighting. LME models indicate similar trajectories over 13 years for both groups, in all measured domains, although patients performed worse across almost all domains at study start. Our sample was diverse both in terms of age and illness duration. Being older at any assessment was associated with worse cognition, and longer illness duration at first assessment had an additional independent negative effect. Longer illness duration likely reflects the impact of several illness-related factors—including prolonged medication exposure, reduced occupational engagement, social network changes, and lifestyle differences—that are difficult to disentangle in an observational design. In sum, our results show that the patient group had lower cognitive performance compared to controls at study start and group differences persisted over the 13-year follow-up.

Our finding of primarily stable cognition that follow broadly the same trajectory as that of controls, aligns with several long-term longitudinal studies following first episode cohorts over 10 years or more.^3^^,^^4^^,^^6^^,^^13^^,^^26^ Fewer studies have followed long-term treated or older patients with schizophrenia for more than a decade. Mohn et al.^12^ followed a community outpatient sample, with similar age profile as our cohort, and found overall stability along with some deterioration in the verbal domain over the 20-year follow-up. Starzer et al.^2^ followed a first episode cohort, with cognitive assessments 10 and 20 years post onset, and similarly found overall stability, though a subgroup of patients showed a declining course. Both studies lacked control groups, making it difficult to assess how their results relate to normative changes with age. Studies following the Suffolk County first admission cohort^8^^,^^42^ observed cognitive decline over 18-25 years, where age-related gaps between patients and controls became more pronounced after age 50. As our sample had a mean age of 55 at the 13-year follow-up, it is possible that even longer observation time would have been needed to observe the divergence reported in these studies. Compared to the Suffolk County cohort, we were able to follow controls longitudinally from baseline onwards, whereas their controls were recruited at the 20-year follow-up, limiting inferences around differential trajectories.

Sensitivity analyses comparing IPW-weighted to unweighted models indicated that, while dropout was non-random in our study, results were substantively similar across weighted and un-weighted models. That our findings remained robust across models lends more confidence to our main finding of stable and non-diverging trajectories in patients and controls. IPW may be even more useful in first episode psychosis cohorts, where bias may operate in either direction, those with severe deficits may dropout to a larger extent (similar to our finding) or those who recover and regain more function may be less likely to prioritize follow-up assessments.

The effect of timepoint was significant in two of the cognitive tasks, working memory, and sustained attention, warranting further discussion. In working memory, all participants performed worse on average after 13 years, irrespective of age at the initial assessment. The non-significant age-coefficient for this domain is notable. Cross-sectional studies in the general population^43^ and in schizophrenia^44^ using the LNS suggest that performance peaks around age 30,^43^ with aging effects becoming more pronounced in the 50s.^44^ It is possible that our linear age term did not fully capture the nonlinear effects and that some of the age-related variance was absorbed in the timepoint coefficient. Another potential explanation would be administration changes at the 13-year follow-up, but no specific changes in test administration were identified, and as the CPI is computerized, there is limited room for variability. Taken together, this findings remains uncertain and should be interpreted with caution.

For the attention domain, the effect over time was the opposite of working memory—both groups, regardless of age at study start, improved performance over time. Improvement in sustained attention is inconsistent with previous cross-sectional literature, which suggests that performance worsens with age in the general population,^45^ as well as in individuals with schizophrenia.^44^ In our model, increasing age was associated with worse performance while the timepoint coefficient was associated with improvement. This suggests that previous test exposure may play a role. Salthouse^46^ has demonstrated that longitudinal data tends to show systematically more positive trajectories than cross-sectional data would, due to practice effects. These effects vary across cognitive domains, likely reflecting differences in how amenable tasks are to employing better strategies,^46^ with tests of memory and reasoning showing larger effects. In the case of CPT-IP, the test can be overwhelming the first time, and one can speculate that, preparedness and/or switching strategy (accuracy vs. speed), may improve later performance. Although changes in test administration cannot be ruled out, the computerized and highly structured nature of the CPT-IP makes this less likely. The parallel improvement in both groups lends further support to the practice effect explanation, although these effects have not been reported on this time scale before.^47^

Results from this study extend those from the 5-year follow-up of this cohort.^31^ The main finding remains the same—patients with long-term treated SSD demonstrate overall cognitive stability over time, with patients consistently performing worse than controls. Furthermore, the cognitive stability observed in our data is consistent with brain imaging findings from this cohort (with approximately 80% participant overlap across timepoints, see Table S18), which showed no significant differences in brain volume loss between patients and controls over the same 13-year period.^29^ This convergence of cognitive and structural findings suggests that long-term treated SSD may not involve progressive neurodegenerative processes beyond normal aging in most patients.

As for specific cognitive domains, Ekerholm et al. reported a trend for controls in our cohort to improve in verbal learning (RAVLT total) at 5-year follow-up, something that was not present in patients.^31^ We failed to replicate this result, although point estimates did suggest an increasing difference between groups over time, where patients declined compared to controls. This pattern should be interpreted cautiously given the lack of statistical significance and the exploratory nature of our analyses. Independent longitudinal studies have identified verbal learning and memory as domains where patients show selective decline over time,^7^^,^^12^^,^^42^ and the present trend, while non-significant, is consistent with that literature. This suggests that verbal learning may represent a domain of specific long-term vulnerability in schizophrenia, warranting further investigation in adequately powered samples.

The 13-year observation period provides evidence that the cognitive stability observed in our sample at 5 years persists over longer follow-up. However, cognitive function is greatly heterogeneous^1^ and this variability has also been found to increase over a 10-year period.^6^ We were unable to model individual trajectories due to sample size constraints, but from visual inspection of Figure 1, it is notable that there is substantial variation in cognitive change over time. One of our aims was to identify predictors of poorer cognitive outcomes at 13 years for those with SSD. We found that higher cognitive function at study start was associated with better cognition at follow-up, but no other predictors were significant. Beneficial or protective effects of cognitive reserve have been observed in previous studies.^25^^,^^26^ We were able to show that this is the case also for a sample of patients with SSD across a wide range of ages and illness durations. Our modest sample size for the analysis of change may have limited our ability to identify additional predictors, although consistent predictors of cognitive change have been difficult to identify also in many previous studies. With more participants, it is possible that pre-morbid IQ would also have been statistically significant as a predictor, as has previously been reported by van Winkel^4^ in first episode patients. Given that baseline cognitive functioning was the primary predictor of 13-year outcome, neuropsychological assessment in the earlier years of illness may have meaningful prognostic value. Individuals with more impairment and less cognitive reserve may have higher long-term risk of decline and, potentially, earlier onset of dementia.^19^ Identifying these individuals earlier on enables clinicians to have informed conversations with patients about their cognitive health and to frame factors such as physical activity, sleep, and cognitive engagement not just in terms of general wellbeing, but also as strategies for preserving cognitive function over time.

In exploratory analyses, we aimed to address the question of accelerated aging, by examining whether age moderated trajectories in the different groups. Previous literature has found evidence of further cognitive deterioration around age 50 years in a large sample^8^ and greater cognitive decline for institutionalized patients over the age of 65 years, when followed over 6 years, compared to those who were younger.^15^ In line with this, the accelerated aging hypothesis suggests that older individuals with SSD in our sample would show greater cognitive decline relative to controls. Our exploratory analyses suggested the opposite pattern across most domains: patient-control trajectory differences tended to be larger in younger individuals and attenuated with increasing age. This pattern reached significance only for the cognitive composite, but did not survive correction for multiple comparisons and was not consistently replicated across individual domains. This pattern should be interpreted cautiously, particularly given the exploratory nature of our study, as several of our findings may be false positives. Furthermore, the age range in our cohort (25-70 years at study start) captures relatively few individuals in the oldest age ranges (>65 years) where accelerated decline might emerge.^15^ Survivor bias may have affected older age groups disproportionately so that the older patients remaining in our sample represent a more resilient or healthy group, beyond what the IPW correction could account for. This type of bias may similarly affect other studies of longitudinal cognition in aging and schizophrenia, contributing to mixed findings in previous studies about whether or not accelerated cognitive decline is present.

### Limitations

Due to the considerable attrition in our sample, we were unable to model random slopes in our LME to further highlight individual differences. It is also important to consider that the study was exploratory in nature—*P* values were left uncorrected and should be interpreted with caution. Our findings should be seen as hypothesis generating and require replication in independent samples. Twelve patients had died by the 13-year follow up, compared to none of the controls, consistent with the well-documented excess mortality in schizophrenia. IPW can only partially address mortality-related attrition, through upweighting survivors with similar baseline characteristics. Given that deceased patients showed poorer cognition and function than survivors at baseline, the likely direction of any residual bias would be toward overestimation of stability. Furthermore, while the definition of dropout used for the IPW (attending any follow-up) was chosen to reflect participants’ ability and motivation to engage with research over time, it may not fully capture missingness specific to the 13-year endpoint.

Participants were selected through convenience sampling, so they were not necessarily representative of the patient population as a whole. Although the majority of patients had a schizophrenia diagnosis, a small proportion had substance induced psychosis or unspecified diagnoses. Furthermore, we were unable to include cumulative medication exposure in our analyses. A strength of the study was that we followed both controls and SSD patients longitudinally, allowing for comparison of practice effects. More formally correcting for practice effects, by using alternate forms or introducing a specific term in our models, was however, beyond the scope of this paper. Although our sample has a wide age range, it is not optimally suited to capture trends in the aging schizophrenia population. Our sample size, and attrition over time, also limited our power to detect small-to-moderate effects.

## Conclusions

This study adds valuable information to the prior knowledge about longitudinal cognitive trajectories in schizophrenia. Cognitive function does appear to remain stable over time, and, although patients had lower cognitive function at study start, the average trajectories for controls and patients in our sample did not differ in slope. Importantly, these findings hold true when we account for dropout of individuals who had worse cognition at study start. There may be domain-specific differences in trajectories, where the verbal domain may be particularly affected in patients. Larger, more diverse samples, and samples focused on advanced age will extend this work on cognitive trajectories in schizophrenia. Higher cognitive function at study start predicted better outcomes at 13 years, highlighting the protective role of cognitive reserve. Early identification and support for individuals with more severe cognitive deficits may be important for optimizing long-term outcomes.

## Supplementary Material

Supplementary_material_Cognitive_Trajectories_13_years_rev2_sbag162
