# Supplementary material for: Cognitive Trajectories over 13 Years in Schizophrenia Spectrum Disorders: Addressing Selective Dropout Bias
Source: Schizophr Bull. 2026 Oct 30;52(6):sbag162. doi: 10.1093/schbul/sbag162 (PMC13720724; doi:10.1093/schbul/sbag162)
Supplement: Supplementary file 1 [file supplementary_material_cognitive_trajectories_13_years_rev2_sbag162.docx]

Supplementary material

**Cognitive Trajectories Over 13 Years in Schizophrenia:**

**Addressing Selective Dropout Bias**

Maria Lee, PhD; Granville J. Matheson, PhD; Ellen E. Lee, MD, PhD; Sara Holmqvist, MSSc; Håkan Nyman, PhD; Ingrid Agartz, MD, PhD; Alison R. Hwong, MD, PhD; Erik G. Jönsson, MD, PhD

Contents:

[Supplementary Methods:	2](#_Toc235440326)

[Age distribution of patients and controls over time	2](#_Toc235440327)

[Figure 1. Age distribution across timepoint for controls and SSD	2](#_Toc235440328)

[Working status	3](#_Toc235440329)

[Table 1. Detailed working status at baseline	3](#_Toc235440330)

[Reasons for dropout	3](#_Toc235440331)

[Table 2. Reasons for dropout	3](#_Toc235440332)

[Table 3. Descriptive comparison of baseline data for patients deceased at 13 year follow up vs those still alive	4](#_Toc235440333)

[Illness duration variable	4](#_Toc235440334)

[Premorbid IQ	4](#_Toc235440335)

[Linear Mixed Effects Models	5](#_Toc235440336)

[Supplementary Results	6](#_Toc235440337)

[Detailed results principal component analysis	6](#_Toc235440338)

[Table. 4 Factor loadings on the first principal component of baseline cognitive data	6](#_Toc235440339)

[Comparisons of LME models without IPW and with IPW	6](#_Toc235440340)

[Table 5. Comparison of LME model without IPW and with IPW: Cognitive composite	6](#_Toc235440341)

[Table 6. Comparison of LME model without IPW and with IPW: Verbal learning	7](#_Toc235440342)

[Table 7. Comparison of LME model without IPW and with IPW: Verbal memory	7](#_Toc235440343)

[Table 8. Comparison of LME model without IPW and with IPW: Working memory	8](#_Toc235440344)

[Table 9. Comparison of LME model without IPW and with IPW: Attention	8](#_Toc235440345)

[Table 10. Comparison of LME model without IPW and with IPW: Processing speed	9](#_Toc235440346)

[Table 11. Comparison of LME model without IPW and with IPW: Cognitive flexibility	9](#_Toc235440347)

[Table 12. Comparison of LME model without IPW and with IPW: Cognitive rigidity, set-shifting failure	10](#_Toc235440348)

[Table 13. Categorical model of poor outcome at 13 years.	10](#_Toc235440349)

[Table 14. Raw scores for those with cognitive data at baseline and 13 year follow up.	10](#_Toc235440350)

[Table 15: Demographic variables complete cases at baseline	11](#_Toc235440351)

[Table 16: Cognitive raw scores for those with complete data at all 3 timepoints	11](#_Toc235440352)

[Table 17: Summary of exploratory analyses of three-way interaction, group*age*time effect (with IPW):	11](#_Toc235440353)

[Table 18: Percentage overlap between MRI cohort and cognition cohort	12](#_Toc235440354)

# Supplementary Methods:

## Age distribution of patients and controls over time

Mean age (SD) at study start was 45.1 (7.4) for controls, 43.4 (9.0) for SSD; at year 5 follow-up it was 48.1 (7.2) for controls and 45.8 (8.0) for SSD, and at year 13 follow-up it was 56.6 (8.3) for controls and 53.4 (8.0) for SSD. Figure 1 depicts the age distribution of participants across the three assessment timepoints.

### Figure 1. Age distribution across timepoint for controls and SSD

## Working status

Working status was originally recorded in several categories: working, unemployment, normal retirement, sickness absence (short term leave from work due to illness), disability pension (long-term/permanent leave from work due to illness). For the demographic comparison this was grouped into a dichotomous variable: Working vs not working. Normal retirement was classified as working in this sample, given that normal retirement indicates a past with working capacity. See Supplementary Table 1 for information on the working status according to detailed categories per group at baseline:

### Table 1. Detailed working status at baseline

|  | Patient | Control |
| --- | --- | --- |
| Working | 25 | 83 |
| Normal retirement | 1 | 0 |
| Unemployed | 1 | 0 |
| Sickness absence | 20 | 2 |
| Disability pension | 54 | 0 |
| NA | 7 | 0 |

## Reasons for dropout

Reasons for drop-out were recorded at the 13-year follow-up when information was available, and are summarized in Table 2.

### Table 2. Reasons for dropout

|  | Patient | Control |
| --- | --- | --- |
| Deceased | 12 | 0 |
| Not contacted (reason not recorded) | 4 | 0 |
| Not contacted due to missing MRI | 18 | 8 |
| Not reachable | 12 | 12 |
| Only MRI at 13 year FU | 2 | 8 |
| Declined | 8 | 1 |
| Declined, due to move | 1 | 2 |
| Declined, displeased with psychiatry | 3 | 0 |
| Physical ailments | 2 | 1 |
| No time | 1 | 7 |
| Abroad | 0 | 1 |
| Total | 63 | 40 |

### Table 3. Descriptive comparison of baseline data for patients deceased at 13 year follow up vs those still alive

| **Baseline demographics** | Patients Alive at 13 Years | N | Patients Deceased at 13 Year | N |
| --- | --- | --- | --- | --- |
| N | 96 |  | 12 |  |
| Participated in 5-year assessment | 51 | 96 | 4 | 12 |
| Age, Years, Mean (SD) | 43.1 (9.2) | 96 | 45.8 (6.2) | 12 |
| Age, Years, Range | 25.7 – 70.3 | 96 | 33.9 – 55.8 | 12 |
| Sex, Female/Male, N | 30 / 66 | 96 | 0 / 12 | 12 |
| Working status, Yes/No, N | 26 / 63 | 89 | 0 / 12 | 12 |
| GAF, score, Mean (SD) | 48.9 (9.5) | 96 | 43.6 (6.7) | 12 |
| Premorbid IQ, Mean (SD) | 89.8 (19.6) | 96 | 83.4 (23.4) | 12 |
| Education, years, Mean (SD) | 12.4 (2.7) | 86 | 10.7 (2.6) | 12 |
| Age of onset, years, Mean (SD) | 24.4 (7.2) | 93 | 27.1 (6.0) | 12 |
| Illness duration, years, Mean (SD) | 18.6 (9.3) | 93 | 18.7 (8.6) | 12 |
| Positive symptoms (SAPS), score, Mean (SD) | 11.7 (9.5) | 54 | 18.5 (11.8) | 4 |
| Negative symptoms (SANS), score, Mean (SD) | 31.0 (21.8) | 55 | 25.0 (15.0) | 4 |
| **Baseline cognition** |  |  |  |  |
| RAVLT Total | 42.6 (12.2) | 96 | 35.0 (10.7) | 12 |
| RAVLT Retention | 8.7 (3.9) | 96 | 6.8 (2.8) | 12 |
| CPT-IP | 0.650 (0.674) | 94 | 0.694 (0.786) | 12 |
| TMT-A | 37.1 (18.1) | 96 | 61.9 (63.2) | 12 |
| TMT-B | 107.9 (72.1) | 96 | 158.4 (139.9) | 12 |
| LNS | 8.7 (3.1) | 96 | 7.4 (3.2) | 12 |
| WCST-PE | 11.9 (7.8) | 96 | 16.4 (11.6) | 12 |

## Illness duration variable

Data on illness duration was not available for some participants. Where possible, age of onset (reported for most individuals) was subtracted from the date of the first assessment and the remaining time was entered as that individual’s illness duration. 3 individuals did not have information on age of onset in any of the interviews or assessments. In those cases, age of onset was imputed using the mice package^1^ and the predictive mean matching (pmm) function in R, using the full dataset as basis for imputation. This imputed age of onset was subtracted from the data of their initial assessment and the remaining time entered as their illness duration.

## Premorbid IQ

Results from the WAIS Information subtest was used as a proxy for pre-morbid IQ. The first available result was used in the analysis, for the vast majority this was completed at study start assessment although some only completed this test at 13 year follow-up.

## Linear Mixed Effects Models

Additional information regarding linear mixed effects models.

Random slopes for participants were attempted, but model failed to converge.

Specification of Linear Mixed Effects model used in final analysis (lmer syntax):

(Cognitive score ~ 1 + Timepoint*Group + Baseline Illness Duration

+ Age (centered) + Sex +

(1 | ID))

Age was centered to avoid potential multicollinearity with illness duration. After centering, variance inflation factors (VIF) for all main predictors were below 5 (group: 4.39, illness duration 4.38, age: 3.45, sex: 1.05), indicating acceptable multicollinearity.

# Supplementary Results

## Detailed results principal component analysis

### Table. 4 Factor loadings on the first principal component of baseline cognitive data

| Cognitive test | PC1 Loading |
| --- | --- |
| RAVLT Total, Verbal learning | -0.418 |
| RAVLT 7, Verbal memory | -0.389 |
| CPT-IP, Attention | -0.289 |
| LNS, Working memory | -0.370 |
| TMT-A (log-reversed), Processing speed | -0.396 |
| TMT-B (log-reversed), Cognitive flexibility | -0.421 |
| WCST Perseverative Errors (log-reversed), Set-shifting | -0.346 |

## Comparisons of LME models without IPW and with IPW

### Table 5. Comparison of LME model without IPW and with IPW: Cognitive composite

| Non-IPW model | | | | IPW-model | | | |
| --- | --- | --- | --- | --- | --- | --- | --- |
| **Fixed Effects** | **Estimate** | **SE** | **p-value** | **Fixed Effects** | **Estimate** | **SE** | **p-value** |
| Intercept | -0.022 | 0.121 | 0.856 | Intercept | -0.020 | 0.130 | 0.878 |
| Group (Control as reference) | -0.868 | 0.251 | 0.0006 *** | Group | -0.906 | 0.261 | 0.0006 *** |
| Timepoint 5 years | 0.153 | 0.117 | 0.191 | Timepoint 5 years | 0.162 | 0.151 | 0.285 |
| Timepoint 13 years | 0.200 | 0.148 | 0.176 | Timepoint 13 years | 0.189 | 0.172 | 0.276 |
| Baseline Illness duration | -0.030 | 0.011 | 0.007 ** | Baseline Illness duration | -0.028 | 0.011 | 0.016 * |
| Age(centered) | -0.034 | 0.009 | 0.0001 *** | Age (centered) | -0.033 | 0.009 | 0.0004*** |
| Sex: Male | -0.103 | 0.130 | 0.428 | Sex: Male | -0.102 | 0.134 | 0.446 |
| Group by Time, Timepoint 5 years: SSD | 0.054 | 0.156 | 0.730 | Group*Time, 5 years: SSD | 0.045 | 0.184 | 0.808 |
| Group by Time, Timepoint 13 years: SSD | -0.108 | 0.141 | 0.446 | Group*Time, 13 years: SSD | -0.051 | 0.166 | 0.760 |
| **Random effects (standard deviations)** | **SD estimate** |  |  | **Random effects** | **SD estimate** |  |  |
| Intercept | 0.738 | - | - | Intercept | 0.699 | - | - |
| Residual variance | 0.499 | - | - | Residual variance | 0.671 | - | - |

Outcome measures in Z-score, i.e. in standard deviation (SD)-units.

### Table 6. Comparison of LME model without IPW and with IPW: Verbal learning

| Non-IPW model | | | | IPW-model | | | |
| --- | --- | --- | --- | --- | --- | --- | --- |
| **Fixed Effects** | **Estimate** | **SE** | **p-value** | **Fixed Effects** | **Estimate** | **SE** | **p-value** |
| Intercept | 56.073 | 1.304 | <.0001 *** | Intercept | 56.169 | 1.373 | <.0001 *** |
| Group (Control as reference) | -7.008 | 2.686 | 0.0097 ** | Group | -6.719 | 2.779 | 0.0164 * |
| Timepoint 5 years | 2.728 | 1.311 | 0.039 * | Timepoint 5 years | 2.789 | 1.515 | 0.065 |
| Timepoint 13 years | 1.238 | 1.618 | 0.444 | Timepoint 13 years | 1.187 | 1.771 | 0.503 |
| Baseline Illness duration | -0.318 | 0.118 | 0.008 ** | Baseline Illness duration | -0.312 | 0.122 | 0.012 * |
| Age(centered) | -0.239 | 0.094 | 0.012 * | Age (centered) | -0.231 | 0.097 | 0.018 * |
| Sex: Male | -3.229 | 1.387 | 0.021 * | Sex: Male | -3.371 | 1.432 | 0.020 * |
| Group by Time, Timepoint 5 years: SSD | -0.809 | 1.761 | 0.647 | Group*Time, 5 years: SSD | -1.535 | 1.820 | 0.400 |
| Group by Time, Timepoint 13 years: SSD | -1.315 | 1.595 | 0.411 | Group*Time, 13 years: SSD | -2.290 | 1.651 | 0.167 |
| **Random effects (standard deviations)** | **SD estimate** |  |  | **Random effects** | **SD estimate** |  |  |
| Intercept | 7.725 | - | - | Intercept | 7.767 | - | - |
| Residual variance | 5.643 | - | - | Residual variance | 6.581 | - | - |

Outcome measure, raw scores on Rey Auditory Verbal Learning Test (RAVLT), trials 1-5. Maximum score 75.

### Table 7. Comparison of LME model without IPW and with IPW: Verbal memory

| Non-IPW model | | | | IPW-model | | | |
| --- | --- | --- | --- | --- | --- | --- | --- |
| **Fixed Effects** | **Estimate** | **SE** | **p-value** | **Fixed Effects** | **Estimate** | **SE** | **p-value** |
| Intercept | 12.688 | 0.406 | <.0001 *** | Intercept | 12.700 | 0.428 | <.0001 *** |
| Group (Control as reference) | -2.687 | 0.835 | 0.0015 ** | Group | -2.815 | 0.861 | 0.001 ** |
| Timepoint 5 years | 0.284 | 0.429 | 0.509 | Timepoint 5 years | 0.292 | 0.492 | 0.553 |
| Timepoint 13 years | 0.269 | 0.517 | 0.604 | Timepoint 13 years | 0.305 | 0.568 | 0.592 |
| Baseline Illness duration | -0.058 | 0.037 | 0.115 | Baseline Illness duration | -0.049 | 0.038 | 0.193 |
| Age(centered) | -0.079 | 0.029 | 0.007 ** | Age (centered) | -0.080 | 0.030 | 0.008 ** |
| Sex: Male | -1.014 | 0.429 | 0.019 * | Sex: Male | -1.044 | 0.442 | 0.019 * |
| Group by Time, Timepoint 5 years: SSD | -0.195 | 0.579 | 0.737 | Group*Time, 5 years: SSD | -0.204 | 0.598 | 0.733 |
| Group by Time, Timepoint 13 years: SSD | -0.640 | 0.525 | 0.224 | Group*Time, 13 years: SSD | -0.781 | 0.545 | 0.154 |
| **Random effects (standard deviations)** | **SD estimate** |  |  | **Random effects** | **SD estimate** |  |  |
| Intercept | 2.339 | - | - | Intercept | 2.332 | - | - |
| Residual variance | 1.845 | - | - | Residual variance | 2.155 | - | - |

Outcome measure, raw score on Rey Auditory Verbal Learning Test (RAVLT), trial 7 (retention, after 30 minutes). Maximum score 15 points.

### Table 8. Comparison of LME model without IPW and with IPW: Working memory

| Non-IPW model | | | | IPW-model | | | |
| --- | --- | --- | --- | --- | --- | --- | --- |
| **Fixed Effects** | **Estimate** | **SE** | **p-value** | **Fixed Effects** | **Estimate** | **SE** | **p-value** |
| Intercept | 10.909 | 0.381 | <.0001 *** | Intercept | 10.893 | 0.394 | <.0001 *** |
| Group (Control as reference) | -1.603 | 0.785 | 0.042 * | Group | -1.615 | 0.516 | 0.043 * |
| Timepoint 5 years | 0.210 | 0.383 | 0.583 | Timepoint 5 years | 0.232 | 0.445 | 0.603 |
| Timepoint 13 years | -1.020 | 0.472 | 0.031 * | Timepoint 13 years | -1.036 | 0.516 | 0.046* |
| Baseline Illness duration | -0.050 | 0.035 | 0.154 | Baseline Illness duration | -0.046 | 0.035 | 0.190 |
| Age(centered) | -0.030 | 0.405 | 0.281 | Age (centered) | -0.028 | 0.028 | 0.315 |
| Sex: Male | 0.039 | 0.404 | 0.923 | Sex: Male | 0.075 | 0.408 | 0.855 |
| Group by Time, Timepoint 5 years: SSD | -0.181 | 0.514 | 0.725 | Group*Time, 5 years: SSD | -0.247 | 0.540 | 0.648 |
| Group by Time, Timepoint 13 years: SSD | -0.608 | 0.465 | 0.193 | Group*Time, 13 years: SSD | -0.716 | 0.490 | 0.146 |
| **Random effects (standard deviations)** | **SD estimate** |  |  | **Random effects** | **SD estimate** |  |  |
| Intercept | 2.256 | - | - | Intercept | 2.176 | - | - |
| Residual variance | 1.646 | - | - | Residual variance | 1.959 | - | - |

Outcome measure, Letter Number Sequencing. Maximum score 21 points.

### Table 9. Comparison of LME model without IPW and with IPW: Attention

| Non-IPW model | | | | IPW-model | | | |
| --- | --- | --- | --- | --- | --- | --- | --- |
| **Fixed Effects** | **Estimate** | **SE** | **p-value** | **Fixed Effects** | **Estimate** | **SE** | **p-value** |
| Intercept | 1.022 | 0.097 | <.0001 *** | Intercept | 1.016 | 0.103 | <.0001 *** |
| Group (Control as reference) | -0.527 | 0.199 | 0.009 ** | Group | -0.563 | 0.203 | 0.006 ** |
| Timepoint 5 years | 0.258 | 0.122 | 0.036 * | Timepoint 5 years | 0.262 | 0.140 | 0.062 |
| Timepoint 13 years | 0.588 | 0.138 | <.0001 *** | Timepoint 13 years | 0.580 | 0.152 | 0.0001*** |
| Baseline Illness duration | -0.002 | 0.008 | 0.772 | Baseline Illness duration | -0.002 | 0.009 | 0.789 |
| Age(centered) | -0.021 | 0.007 | 0.002 ** | Age (centered) | -0.021 | 0.007 | 0.003 ** |
| Sex: Male | 0.159 | 0.100 | 0.115 | Sex: Male | 0.170 | 0.102 | 0.097 |
| Group by Time, Timepoint 5 years: SSD | 0.138 | 0.167 | 0.409 | Group*Time, 5 years: SSD | 0.086 | 0.173 | 0.618 |
| Group by Time, Timepoint 13 years: SSD | 0.169 | 0.155 | 0.277 | Group*Time, 13 years: SSD | 0.257 | 0.160 | 0.109 |
| **Random effects (standard deviations)** | **SD estimate** |  |  | **Random effects** | **SD estimate** |  |  |
| Intercept | 2.256 | - | - | Intercept | 0.454 | - | - |
| Residual variance | 1.646 | - | - | Residual variance | 0.641 | - | - |

Outcome measure; Continuous Performance Test – Identical Pairs (CPT-IP), d’. Plausible range: -4.24 to +4.24

### Table 10. Comparison of LME model without IPW and with IPW: Processing speed

| Non-IPW model | | | | IPW-model | | | |
| --- | --- | --- | --- | --- | --- | --- | --- |
| **Fixed Effects** | **Estimate** | **SE** | **p-value** | **Fixed Effects** | **Estimate** | **SE** | **p-value** |
| Intercept | 3.152 | 0.049 | <.0001 *** | Intercept | 3.148 | 0.052 | <.0001 *** |
| Group (Control as reference) | 0.291 | 0.100 | 0.004 ** | Group | 0.283 | 0.104 | 0.007 ** |
| Timepoint 5 years | 0.035 | 0.059 | 0.546 | Timepoint 5 years | 0.041 | 0.068 | 0.548 |
| Timepoint 13 years | -0.089 | 0.067 | 0.182 | Timepoint 13 years | -0.070 | 0.075 | 0.349 |
| Baseline Illness duration | 0.010 | 0.004 | 0.025 * | Baseline Illness duration | 0.009 | 0.005 | 0.038 * |
| Age(centered) | 0.016 | 0.003 | <.0001 *** | Age (centered) | 0.014 | 0.003 | <.0001 *** |
| Sex: Male | -0.017 | 0.051 | 0.735 | Sex: Male | -0.014 | 0.052 | 0.793 |
| Group by Time, Timepoint 5 years: SSD | -0.062 | 0.080 | 0.438 | Group*Time, 5 years: SSD | -0.045 | 0.083 | 0.587 |
| Group by Time, Timepoint 13 years: SSD | 0.011 | 0.072 | 0.876 | Group*Time, 13 years: SSD | 0.038 | 0.076 | 0.647 |
| **Random effects (standard deviations)** | **SD estimate** |  |  | **Random effects** | **SD estimate** |  |  |
| Intercept | 0.255 | - | - | Intercept | 0.250 | - | - |
| Residual variance | 0.260 | - | - | Residual variance | 0.307 | - | - |

Outcome measure Trail Making Test A (TMT-A), time in seconds.

### Table 11. Comparison of LME model without IPW and with IPW: Cognitive flexibility

| Non-IPW model | | | | IPW-model | | | |
| --- | --- | --- | --- | --- | --- | --- | --- |
| **Fixed Effects** | **Estimate** | **SE** | **p-value** | **Fixed Effects** | **Estimate** | **SE** | **p-value** |
| Intercept | 4.014 | 0.059 | <.0001 *** | Intercept | 4.010 | 0.060 | <.0001 *** |
| Group (Control as reference) | 0.349 | 0.121 | 0.004 ** | Group | 0.341 | 0.122 | 0.006 ** |
| Timepoint 5 years | 0.038 | 0.051 | 0.459 | Timepoint 5 years | 0.034 | 0.061 | 0.541 |
| Timepoint 13 years | -0.067 | 0.069 | 0.336 | Timepoint 13 years | -0.055 | 0.075 | 0.463 |
| Baseline Illness duration | 0.010 | 0.005 | 0.053 | Baseline Illness duration | 0.011 | 0.005 | 0.053 |
| Age(centered) | 0.015 | 0.004 | 0.0004 *** | Age (centered) | 0.014 | 0.004 | 0.001 ** |
| Sex: Male | 0.097 | 0.063 | 0.125 | Sex: Male | 0.099 | 0.063 | 0.117 |
| Group by Time, Timepoint 5 years: SSD | 0.016 | 0.068 | 0.813 | Group*Time, 5 years: Schizophrenia | 0.000 | 0.073 | 0.998 |
| Group by Time, Timepoint 13 years: Schizophrenia | 0.071 | 0.062 | 0.250 | Group*Time, 13 years: Schizophrenia | 0.079 | 0.067 | 0.239 |
| **¢Random effects (standard deviations)** | **SD estimate** |  |  | **Random effects** | **SD estimate** |  |  |
| Intercept | 0.369 | - | - | Intercept | 0.357 | - | - |
| Residual variance | 0.215 | - | - | Residual variance | 0.263 | - | - |

Outcome measure Trail Making Test A (TMT-B), log-units (seconds)

### Table 12. Comparison of LME model without IPW and with IPW: Cognitive rigidity, set-shifting failure

| Non-IPW model | | | | IPW-model | | | |
| --- | --- | --- | --- | --- | --- | --- | --- |
| **Fixed Effects** | **Estimate** | **SE** | **p-value** | **Fixed Effects** | **Estimate** | **SE** | **p-value** |
| Intercept | 1.982 | 0.073 | <.0001 *** | Intercept | 1.979 | 0.079 | <.0001 *** |
| Group (Control as reference) | 0.274 | 0.151 | 0.070 | Group | 0.281 | 0.158 | 0.077 |
| Timepoint 5 years | -0.141 | 0.091 | 0.121 | Timepoint 5 years | -0.142 | 0.103 | 0.171 |
| Timepoint 13 years | -0.215 | 0.101 | 0.035 * | Timepoint 13 years | -0.220 | 0.113 | 0.051 |
| Baseline Illness duration | 0.012 | 0.007 | 0.074 | Baseline Illness duration | 0.011 | 0.007 | 0.104 |
| Age(centered) | 0.018 | 0.005 | 0.0006 *** | Age (centered) | 0.018 | 0.005 | 0.001 ** |
| Sex: Male | -0.104 | 0.076 | 0.176 | Sex: Male | -0.097 | 0.080 | 0.226 |
| Group by Time, Timepoint 5 years: Schizophrenia | -0.090 | 0.123 | 0.466 | Group*Time, 5 years: Schizophrenia | -0.055 | 0.126 | 0.664 |
| Group by Time, Timepoint 13 years: Schizophrenia | 0.138 | 0.113 | 0.224 | Group*Time, 13 years: Schizophrenia | 0.173 | 0.115 | 0.134 |
| **Random effects (standard deviations)** | **SD estimate** |  |  | **Random effects** | **SD estimate** |  |  |
| Intercept | 0.372 | - | - | Intercept | 0.383 | - | - |
| Residual variance | 0.402 | - | - | Residual variance | 0.462 | - | - |

Outcome measure Wisconsin Card Sorting Test (WCST) Perseverative Errors (in log-units).

### Table 13. Categorical model of poor outcome at 13 years.

| Predictor | Estimate (β) | Std. Error | z value | *p* value | 95 % CI |
| --- | --- | --- | --- | --- | --- |
| (Intercept) | 5.129 | 3.16 | 1.62 | 0.105 | -0.65 – 12.16 |
| Age of onset | -0.071 | 0.08 | -0.85 | 0.395 | -0.25 – 0.09 |
| Sex (Male) | -1.084 | 0.89 | -1.23 | 0.221 | -2.88 – 0.67 |
| Baseline cognition | -0.044 | 0.59 | -0.08 | 0.941 | -1.19 – 1.18 |
| Premorbid IQ | -0.410 | 0.03 | -1.59 | 0.113 | -0.10 - 0.01 |

### Table 14. Raw scores for those with cognitive data at baseline and 13 year follow up.

| Cognitive test | SSD Baseline  Mean (SD) N | SSD Follow up  Mean (SD) N | *p* | Controls Baseline  Mean (SD) N | Controls  Follow up  Mean (SD) N | *p* | *p* Patient vs HC Baseline | *p* Patient vs HC 13 year |
| --- | --- | --- | --- | --- | --- | --- | --- | --- |
| RAVLT Total | 47.3 (10.2) 45 | 43.3 (10.5) 44 | 0.0695 | 55.7 (6.1) 45 | 53.6 (9.5) 45 | 0.2295 | **<0.0001** | **<0.0001** |
| RAVLT 7 | 9.8 (3.5) 45 | 8.1 (3.4) 44 | **0.0265** | 12.6 (2) 45 | 11.8 (2.5) 43 | 0.1316 | **<0.0001** | **<0.0001** |
| CPT-IP *d’* | 0.644 (0.699) 44 | 1.162 (0.788) 41 | **0.0020** | 1.218 (0.686) 41 | 1.485 (0.865) 43 | 0.1142 | **0.00018** | 0.0778 |
| TMT-A | 30.6 (9.5) 45 | 36.6 (13.7) 44 | **0.0187** | 24.8 (8.3) 45 | 26.8 (9.0) 44 | 0.2694 | **0.0027** | **0.0001** |
| TMT-B | 79.5 (28.0) 45 | 102.6 (48.2) 43 | **0.0081** | 58.5 (18.1) 45 | 67.8 (23.7) 44 | **0.0400** | **<0.0001** | **<0.0001** |
| LNS | 9.8 (2.4) 45 | 7.5 (2.6) 44 | **<0.0001** | 11.2 (2.2) 45 | 9.7 (2.9) 45 | **0.0079** | **0.00458** | **0.0003** |
| WCST-PE | 10.1 (7.5) 45 | 12.2 (7.9) 42 | **0.0067** | 7.2 (4.2) 45 | 7.4 (4.5) 44 | 0.8225 | **0.02561** | **0.0009** |

SSD (Schizophrenia Spectrum Disorder), RAVLT (Rey Auditory Verbal Learning Test) Total (Verbal learning), RAVLT 7 (Verbal memory), CPT-IP (Continuous Performance Test – Identical Pairs) (Attention and Vigilance), TMT-A (Trail Making Test-A, Processing speed), TMT-B (Cognitive flexibility), LNS (Letter Number Sequencing) (Working memory), WCST-PE (Wisconsin Card Sorting Test – Perseverative Errors) (Set-shifting)

### Table 15: Demographic variables complete cases at baseline

|  | Patients (N= 26) | Controls (N= 24) | t / χ^2^ | Df | p |
| --- | --- | --- | --- | --- | --- |
| Age, mean (SD), years | 39.7 (8.0) | 43.1 (7.4) | 1.52 | 48.0 | 0.134 |
| Age, range, year | 25 – 52 | 27 – 51 |  |  |  |
| Sex, F/M, N (%) | 3 (12)/ 23 (88) | 11 (46)/13 (54) |  |  | 0.011 |
| Working status, Yes/No | 15 / 11 | 23 / 1 |  |  | <0.001 |
| Education, mean (SD), year | 13.0 (2.1) N = 25 | 13.7 (2.6) | 0.93 | 44.14 | 0.359 |
| GAF, mean (SD), score | 49.6 (10.9) N = 22 | 87.4 (5.4) N = 23 | 14.65 | 30.4 | <0.001 |
| Diagnosis, N (%) |  |  |  |  |  |
| Schizophrenia | 18 (69) |  |  |  |  |
| Schizoaffective disorder | 4 (15) |  |  |  |  |
| Psychotic disorder NOS | 3 (12) |  |  |  |  |
| Substance-induced | 1 (4) |  |  |  |  |
| Age of onset, mean (SD), year | 23.0 (4.2) N = 24 |  |  |  |  |
| Age of onset, range, year | 20 – 26 |  |  |  |  |
| Duration of illness, mean (SD), year | 16.5 (8.8) |  |  |  |  |
| Duration of illness, range, year | 1 – 36 |  |  |  |  |
| Medication status N, (%) |  |  |  |  |  |
| Antipsychotics use | 23 (88) |  |  |  |  |
| No antipsychotics | 3 (12) |  |  |  |  |

### Table 16: Cognitive raw scores for those with complete data at all 3 timepoints

| Cognitive test | Patient Baseline  Mean (SD) (N) | Patient  5 year  Mean (SD) (N) | Patient  13 year  Mean (SD) (N) | Controls Baseline  Mean (SD) (N) | Controls 5 year  Mean (SD) (N) | Controls  13 year  Mean(SD) (N) |
| --- | --- | --- | --- | --- | --- | --- |
| RAVLT Total | 47.5 (9.3) | 48.6 (8.9) | 44.5 (10.0) | 56.8 (6.3) | 58.8 (6.7) | 56.2 (9.7) |
| RAVLT 7 | 10.0 (3.1) | 9.4 (3.0) | 8.4 (3.6) | 12.5 (2.1) 25 | 12.8 (2.3) | 11.9 (2.5) 23 |
| CPT-IP *d’* | 0.850 (0.680) | 1.150 (0.812) | 1.344 (0.775) 23 | 1.350 (0.770) | 1.429 (1.026) | 1.549 (0.883) 22 |
| TMT-A | 28.9 (7.6) | 31.0 (13.7) | 33.9 (14.2) | 23.5 (8.6) | 25.0 (6.4) | 26.3 (9.4) |
| TMT-B | 75.3 (29.6) | 94.0 (41.7) | 93.4 (30.9) 25 | 57.8 (21.0) | 64.1 (29.4) | 62.7 (21.5) |
| LNS | 10.1 (2.2) | 9.7 (2.7) | 7.9 (2.3) | 11.8 (2.4) | 11.3 (2.5) | 9.7 (3.4) |
| WCST-PE | 10.0 (6.9) | 8.1 (5.6) | 9.9 (7.0) 24 | 8.1 (5.4) | 6.5 (4.2) | 7.2 (4.4) 23 |

N, unless otherwise specified, is 26 for patients and 24 for controls

### Table 17: Summary of exploratory analyses of three-way interaction, group*age*time effect (with IPW):

| **Cognitive domain** | **Estimate group*age*time13** | ***p*** | **Adjusted *p**** | **Statistically better fit?**** |
| --- | --- | --- | --- | --- |
| Composite | +0.048 | 0.022 | 0.176 | No |
| Verbal learning (RAVLT Total) | +0.044 | 0.829 | 0.829 | No |
| Verbal memory (RAVLT, trial 7) | +0.106 | 0.120 | 0.320 | No |
| Working memory (LNS) | +0.066 | 0.290 | 0.464 | No |
| Attention (CPT-IP) | +0.025 | 0.207 | 0.414 | No |
| Processing speed (TMT-A) | -0.017 | 0.073 | 0.292 | No |
| Processing speed, cognitive flexibility (TMT-B) | -0.005 | 0.546 | 0.728 | No |
| Set-shifting problems (WCST, perseverative errors) | -0.003 | 0.825 | 0.829 | No |

* False Discovery Rate (FDR) correction using Benjamini-Hochberg method

**Using AIC/BIC criteria

### Table 18: Percentage overlap between MRI cohort and cognition cohort

| Timepoint | Total | Patients | Controls |
| --- | --- | --- | --- |
| Baseline | 86 % | 89 % | 83 % |
| 5-year follow-up | 68 % | 74 % | 63 % |
| 13-year follow-up | 87 % | 94 % | 82 % |

References

1.	Buuren S van, Groothuis-Oudshoorn K. mice: Multivariate Imputation by Chained Equations in R. *J Stat Softw*. 2011;45(3). doi:10.18637/jss.v045.i03
